# Leukocyturia and hematuria enable non-invasive differentiation of Bowman’s capsule rupture severity in PR3-ANCA glomerulonephritis

**DOI:** 10.1007/s40620-022-01486-8

**Published:** 2022-12-21

**Authors:** Eva Baier, Ingmar Alexander Kluge, Samy Hakroush, Désirée Tampe, Björn Tampe

**Affiliations:** 1 Department of Nephrology and Rheumatology, University Medical Center Göttingen, Göttingen, Germany; 2 Institute of Pathology, University Medical Center Göttingen, Göttingen, Germany; 3 SYNLAB Pathology Hannover, SYNLAB Holding Germany, Augsburg, Germany

**Keywords:** Renal vasculitis, Leukocyturia, Hematuria, Bowman’s capsule rupture

## Abstract

**Background:**

Renal involvement is a common and severe complication of anti-neutrophil cytoplasmic antibody-(ANCA)-associated vasculitis potentially resulting in pauci-immune necrotizing and crescentic ANCA glomerulonephritis (GN) with rapid deterioration of kidney function, progression to end stage kidney disease or, if left untreated, lethal exitus. Analysis of the urinary sediment routinely supports clinical management of ANCA GN, but histopathological implications of aberrancies in the urinary sediment mostly remain elusive. Therefore, we aimed to systematically assess the correlation of aberrancies in the urinary sediment and clinico-pathologic findings.

**Methods:**

A total of 42 kidney biopsies with ANCA GN were retrospectively analyzed in a single-center observational study. Laboratory and histopathological parameters were systematically analyzed and correlated with findings of the urinary sediment.

**Results:**

In the overall ANCA GN cohort, leukocyturia and hematuria were associated among each other, and with markers for non-selective glomerular damage, respectively. Non-invasive measurement of leukocyturia indicated focal (but not extensive) Bowman’s capsule rupture (BCR) specifically in proteinase-3 (PR3)-ANCA GN, whereas hematuria correlated with extensive (but not focal) BCR. Concerning intrarenal immune cell infiltration, leukocyturia was associated with tubulointerstitial plasma cell infiltration in PR3-ANCA GN. Finally, none of these associations were detectable in myeloperoxidase-ANCA GN, implying different modes of kidney damage.

**Conclusion:**

We herein expand our current knowledge by providing evidence that leukocyturia and hematuria enable non-invasive differentiation of BCR severity specifically in PR3-ANCA GN.

**Graphical abstract:**

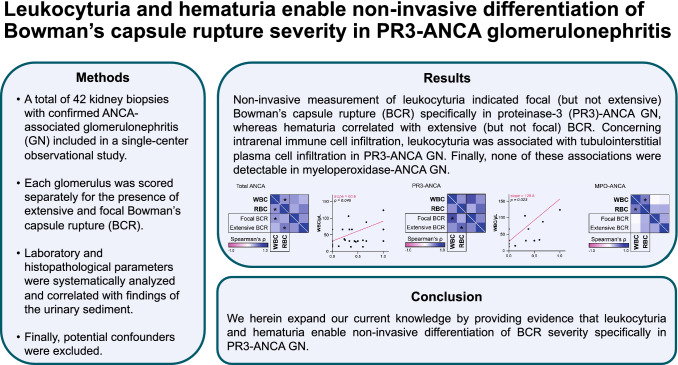

**Supplementary Information:**

The online version contains supplementary material available at 10.1007/s40620-022-01486-8.

## Introduction

Anti-neutrophil cytoplasmic antibody (ANCA)-associated vasculitis (AAV) is a small vessel vasculitis most frequently presenting as microscopic polyangiitis or granulomatosis with polyangiitis according to the 2012 revised Chapel Hill Consensus Conference Nomenclature of Vasculitides [1, 2]. Renal involvement is a common and severe complication of AAV, potentially resulting in pauci-immune necrotizing and crescentic ANCA glomerulonephritis (GN) with rapid deterioration of kidney function, progression to end stage kidney disease or, if left untreated, lethal exitus [2]. Clinico-pathologic studies by the European Vasculitis Study Group (EUVAS) demonstrated that distinct glomerular lesions are related to renal outcome in ANCA GN [3–6]. We and others recently reported that Bowman’s capsule rupture (BCR) links glomerular damage to tubulointerstitial inflammation in a considerable subset of patients with ANCA GN [7–10]. Particularly, an increased fraction of glomeruli affected by extensive BCR in ANCA GN was associated with tubulointerstitial inflammation, suggesting that interstitial inflammation may also have predictive value in assessing the risk for decline of kidney function in ANCA GN [9]. Furthermore, focal BCR with less extensive lesions was observed even more frequently in ANCA GN [8–11]. In this context, it was proposed that the fibrous strand-strengthened basement membrane of the Bowman’s capsule might serve as a barrier for inflammatory cell invasion into Bowman’s space [12]. Therefore, an intact Bowman’s capsule prevents inflammatory cells from gaining access to the glomerular space, but once Bowman’s capsule is breached, inflammatory cells can access the glomerular space in crescentic GN with BCR enabling direct pathological interaction between both compartments [12]. Moreover, different organizational clusters of lymphocytic infiltrates in ANCA GN were found to influence renal outcome and renal relapse after induction therapy [13, 14]. We previously reported on different subsets of intrarenal immune cell subtypes, such as neutrophils, eosinophils, plasma cells, and mononuclear cell infiltrates (macrophages, lymphocytes) that contribute to inflammation and renal injury in ANCA GN via distinct pathogenetic mechanisms, wherein correlative analyses revealed a linkage between the extent of BCR and distinct immune cell subtypes [10]. Despite growing knowledge about these histopathological findings, association with aberrancies of the urinary sediment and particularly leukocyturia remains elusive. Therefore, we aimed to systematically assess the correlation of aberrancies in the urinary sediment and clinico-pathologic findings.

## Methods

### Study population

A total of 42 cases of biopsy-proven ANCA GN diagnosed between 2015 and 2020 at the University Medical Center Göttingen, Germany were retrospectively included. The patient cohort has previously been partly described [9, 10]. The studies involving human participants were reviewed and approved by the Institutional Review Board of the University Medical Center Göttingen, Germany (no. 4/8/19). The patients/participants provided written informed consent for the use of routinely collected data for research purposes as part of their regular medical care in the contract of the University Medical Center Göttingen. Medical records were used to obtain data on age, sex, medication, treatment received, onset of symptoms, date of admission and kidney biopsy, laboratory results and urin analysis. Stages of chronic kidney disease before admission were assessed based on Kidney Disease: Improving Global Outcomes (KDIGO) guidelines [15].

### Urin analysis

Analysis of the urinary sediment was performed by the automated urine analyzer cobas® 6500 by Roche Diagnostics GmbH. The assessment of leukocytes (white blood cells, [WBCs]), erythrocytes (red blood cells, [RBCs]), squamous epithelial cells, hyaline casts, granular casts, and bacteria with conversion from ‘particles per high power field’ into ‘particles per µL’ was performed according to the manufacturer’s protocol. Additionally, analysis of urinary dipstick was carried out with the manufacturer’s conversion table. Leukocyturia was defined as more than 20 WBCs per µL urine [16]. Hematuria was regarded as more than ten RBCs per µL urine [16]. Cases of leukocyturia and coincident bacteriuria in the urine culture were excluded from analysis. Automated count of dysmorphic RBCs was excluded from analysis based on scientific evidence of dysmorphic RBCs being underreported by automated analysis using cobas® 6500 by Roche Diagnostics GmbH [17]. Acanthocytes were also excluded from analysis.

### Renal histopathology

A renal pathologist (SH) evaluated all biopsies, blinded to clinical data and analysis. Within every renal biopsy specimen, each glomerulus was scored separately for the presence of necrosis, crescents, and global sclerosis. Consequently, the percentage of glomeruli with any of these features was calculated as a fraction of the total number of glomeruli in each renal biopsy. As recently described, each glomerulus was scored separately for the presence of BCR in injured glomeruli (crescentic and/or necrotic). Subsequently, the percentage of glomeruli affected by BCR was calculated as a fraction of the total number of glomeruli in each renal biopsy [9, 10]. Based on these scorings, histopathological subgrouping according to Berden et al. (focal, crescentic, mixed or sclerotic class) and Brix et al. (high risk, medium risk, low risk) was performed [18, 19]. Renal biopsies were also evaluated analogous to the Banff score for allograft pathology [20]. Systematic histological scoring of tubular injury lesions was evaluated as previously described [21]**.** In addition, infiltrates of neutrophils, eosinophils, plasma cells, and mononucleated cells (macrophages, lymphocytes) were quantified as a fraction of the area of total cortical inflammation as previously described [22].

### Statistical methods

Variables were tested for normal distribution using the Shapiro–Wilk test. Statistical comparisons were not formally powered or prespecified. Continuous and ordinal variables were presented as mean ± standard deviation, categorical variables as percentages of total. Comparison of means between groups was conducted with the unpaired Student’s t-test in case of normally distributed values and with the Mann–Whitney test in case of non-normally distributed values. Non-parametric between-group-comparisons were conducted with Pearson’s Chi-square test. Spearman's correlation was performed to assess correlations and heatmaps reflect the mean values of Spearman's *ρ*, asterisks indicate statistically significant correlations. Probability values (*p* values) below 0.05 were considered statistically significant. Data analyses were performed with GraphPad Prism (version 9.3.1 for MacOS, GraphPad Software, San Diego, California, USA) and IBM SPSS Statistics (version 27 for MacOS, IBM Corporation, Armonk, NY, USA).

## Results

### Correlation of urinary sediment components in ANCA GN

**Fig. 1:**
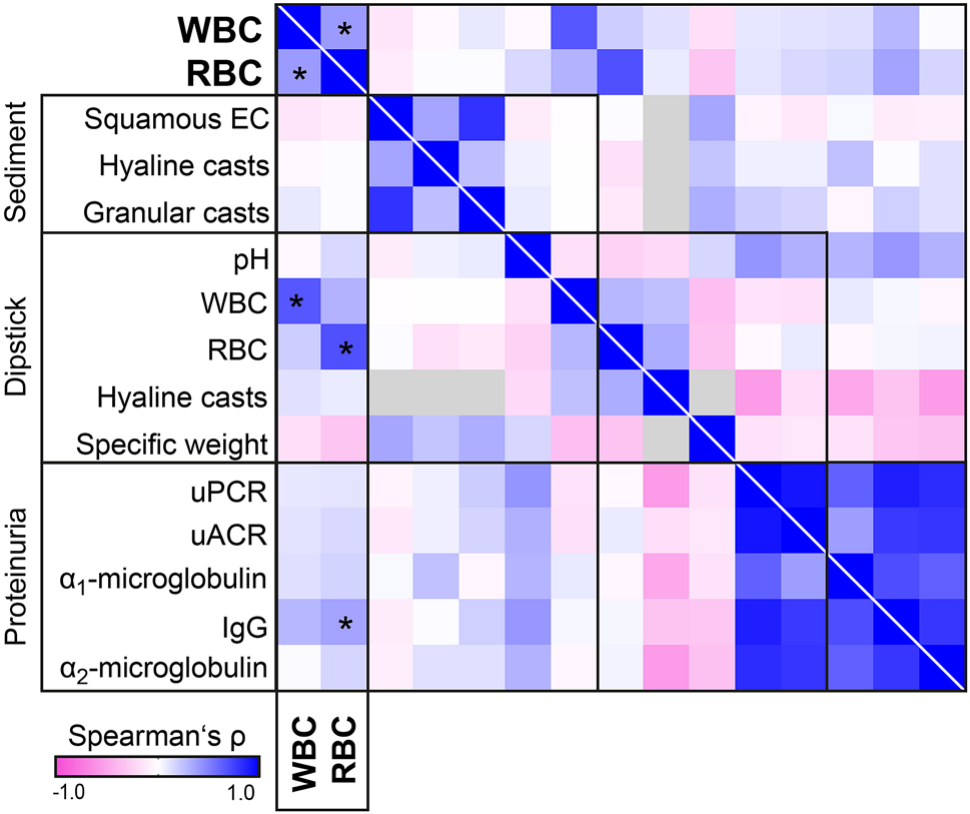
Correlative analysis of urinary findings in ANCA GN. Association of leukocyturia (WBCs) and hematuria (RBCs) with urinary sediment, dipstick and proteinuria; proteinuric findings were standardized per g creatinine; cellular components were quantified in particles per µL; heatmap visualizes mean values of Spearman’s ρ; asterisks represent *p* < 0.05, gray lines within each heatmap reflect no data analysis due to absence of the respective parameter. *ANCA* anti-neutrophil cytoplasmic antibody, *dip. RBCs* red blood cells from dipstick, *dip. WBCs* white blood cells from dipstick, *ECs* epithelial cells, *GN* glomerulonephritis, *IgG* immunoglobulin G per creatinine (mg/g), *RBCs* red blood cells, *uACR* urinary albumin-to-creatinine ratio, *uPCR* urinary protein-to-creatinine ratio, *WBCs* white blood cells, *α*_*1*_*-microglobulin* α_1_-microglobulin per creatinine (mg/g), *α*_*2*_*-microglobulin* α_2_-microglobulin per creatinine (mg/g)

After excluding 5 cases with leukocyturia and coincident bacteriuria in the urine culture, a total of 42 urine samples from patients with biopsy-proven ANCA GN were included (Supplemental Fig. 1). The baseline characteristics of the overall cohort are shown in Supplemental Table 1. Twenty-one/42 (50.0%) were positive for proteinase 3 (PR3)-ANCA, 21/42 (50.0%) were positive for myeloperoxidase (MPO)-ANCA, while there were no cases of double-positivity. We first analyzed the association of urinary sediment components among each other. Correlative analysis revealed that leukocyturia significantly correlated with hematuria (*p* = 0.011, *r* = 0.391, Fig. 1). Other urinary sediment components of human origin, namely squamous epithelial cells, hyaline casts, and granular casts showed no relevant correlation with leukocyturia or hematuria (Fig. 1). Regarding urinary dipstick findings, WBCs and RBCs correlated and confirmed findings by urinary sediment for WBCs (*p* < 0.005, *r* = 0.657) and RBCs (*p* < 0.005, *r* = 0.686, Fig. 1). Correlative analyses of proteinuria revealed a significant association of urinary IgG levels with hematuria (*p* = 0.020, *r* = 0.359, Fig. 1), implying non selective glomerular damage.

### Leukocyturia correlates with distinct histopathological lesions differing between PR3-ANCA and MPO-ANCA GN

**Fig. 2:**
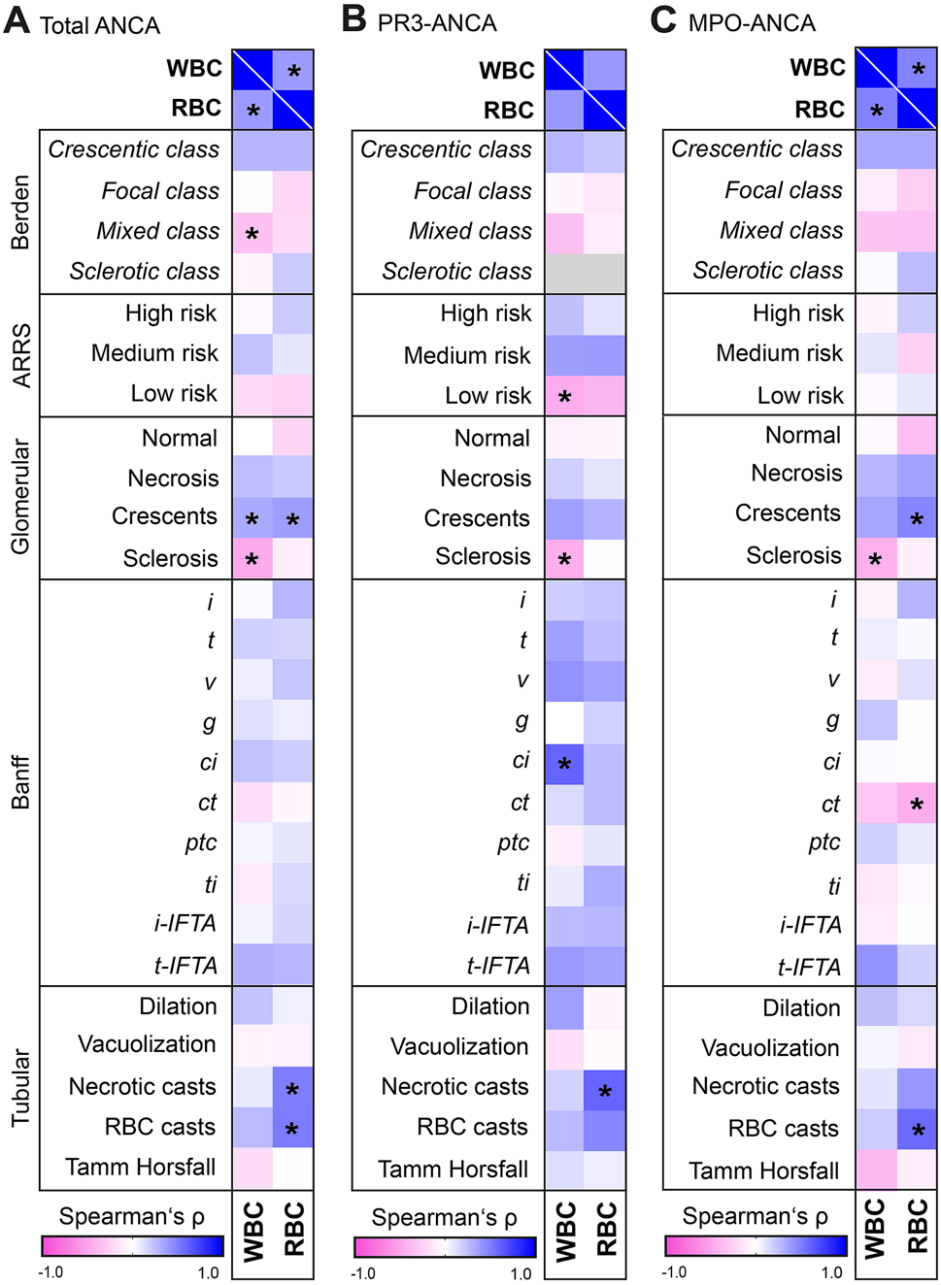
Association of leukocyturia and hematuria with histopathological findings. **A**–**C** WBCs indicate leukocyturia, RBCs represent hematuria, histopathological findings feature Berden classification, ARRS, glomerular lesions as quantified in percentage of total, Banff scoring and tubular lesions. Heatmap visualizes mean values of Spearman’s ρ, asterisks represent *p* < 0.05; gray lines within the heatmap reflect no data analysis because of no sclerotic class in PR3-ANCA GN. *ANCA* anti-neutrophil cytoplasmic antibody, *ARRS* ANCA renal risk score, *ci* interstitial fibrosis, *ct* tubular atrophy, *g* glomerulitis, *GN* glomerulonephritis, *i* interstitial inflammation, *i-IFTA* inflammation in areas of IFTA, *MPO* myeloperoxidase, *PR3* proteinase 3, *ptc* peritubular capillaritis, *RBCs* red blood cells, *t* tubulitis, *ti* total inflammation, *t-IFTA* tubulitis in areas of IFTA, *v* intimal arteritis, *WBCs* white blood cells

Next, we analyzed the association of distinct histopathological findings focusing on leukocyturia and hematuria for the overall cohort of ANCA GN, and then subdivided by PR3-ANCA and MPO-ANCA GN. In the overall cohort, leukocyturia positively correlated with the fraction of glomerular crescent formation (*p* = 0.033, *r* = 0.330), but also offered an inverse association with the fraction of glomerular sclerosis (*p* = 0.001, *r* =  − 0.483, Fig. 2A), both features that mainly constitute “mixed” class ANCA GN, which further featured an inverse association with leukocyturia (*p* = 0.020, *r* =  − 0.357, Fig. 2A). Regarding the ANCA renal risk score (ARRS), leukocyturia inversely correlated with low risk specifically in PR3-ANCA GN (*p* = 0.029, *r* =  − 0.475, Fig. 2A–C). The inverse association of leukocyturia and the fraction of glomerular sclerosis was maintained in both, PR3-ANCA GN (*p* = 0.034, *r* =  − 0.465, Fig. 2B) and MPO-ANCA GN (*p* = 0.044, *r* =  − 0.443, Fig. 2C). Hematuria was associated with the fraction of glomerular crescent formation (*p* = 0.013, *r* = 0.379, Fig. 2A), specifically observed in MPO-ANCA GN (*p* = 0.031, *r* = 0.472, Fig. 2B, C). Screening of distinct histopathological lesions according to the Banff classification revealed no correlation with leukocyturia in the overall ANCA GN cohort (Fig. 2A). Particularly, features of tubulointerstitial inflammation including tubulitis (*t*) and interstitial inflammation (*i*) showed no significant association with leukocyturia (*p* = 0.261 and *p* = 0.936, respectively, Fig. 2A). In contrast, leukocyturia was significantly associated with interstitial fibrosis (*ci*) in PR3-ANCA GN (*p* = 0.004, *r* = 0.605, Fig. 2B), while tubular atrophy (*ct*) inversely correlated with hematuria specifically in MPO-ANCA GN (*p* = 0.039, *r* =  − 0.464, Fig. 2C). Regarding tubular lesions, hematuria correlated with necrotic casts in PR3-ANCA (*p* = 0.012, *r* = 0.601, Fig. 2B), and with RBC casts in MPO-ANCA GN (*p* = 0.008, *r* = 0.577, Fig. 2C), while both associations were observable in the overall ANCA GN cohort (Fig. 2A).

### Leukocyturia is correlated with focal Bowman’s capsule rupture specifically in PR3-ANCA GN

**Fig. 3:**
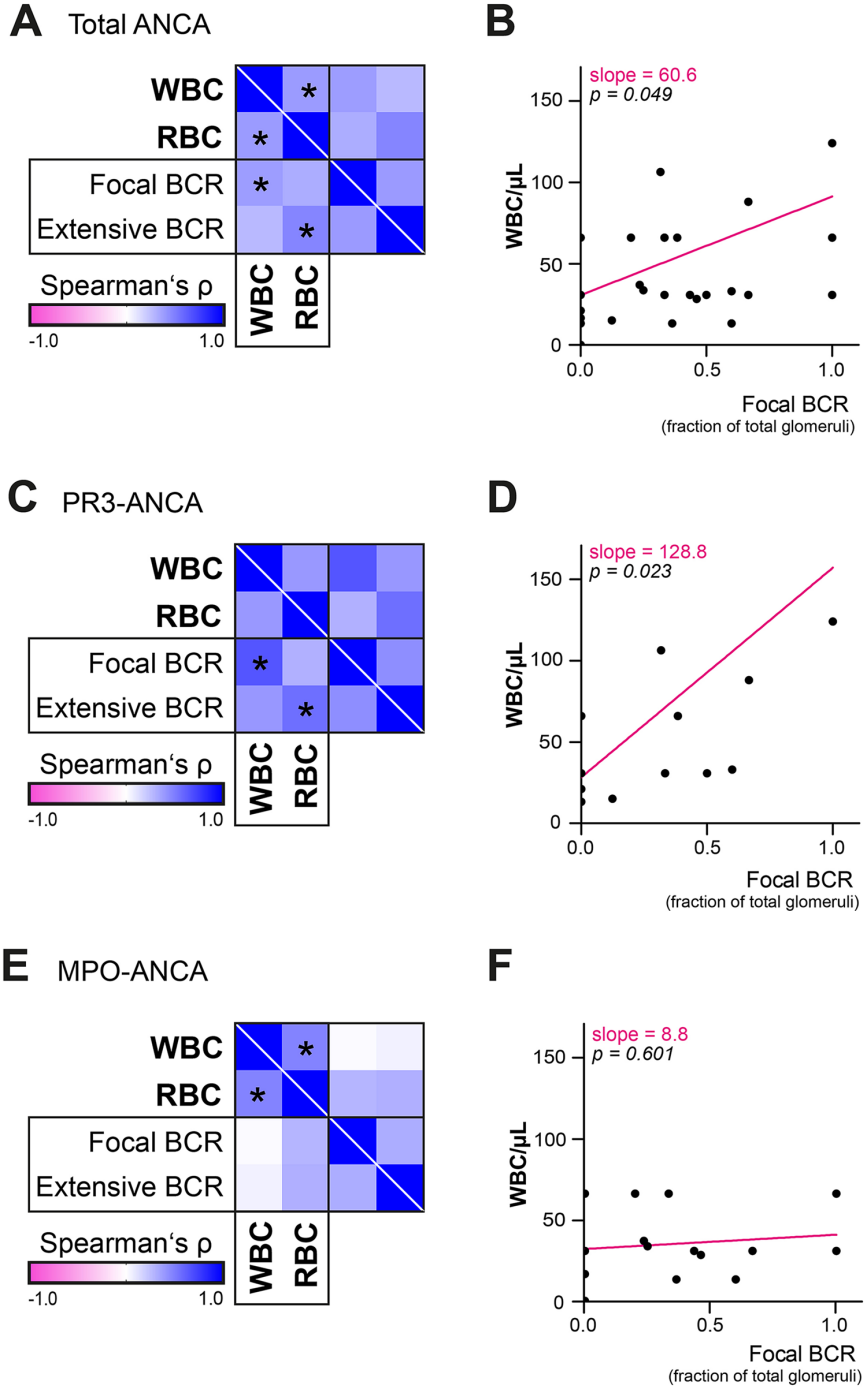
Correlation of leukocyturia and hematuria with BCR. **A**–**F** WBCs indicate leukocyturia, RBCs represent hematuria, BCR was categorized as “focal” and “extensive”, indicated as fraction of total amount of glomeruli. Heatmap visualizes mean values of Spearman’s ρ; asterisks represent *p* < 0.05, regression lines are shown in magenta. *ANCA* anti-neutrophil cytoplasmic antibody, *BCR* Bowman’s capsule rupture, *GN* glomerulonephritis, *MPO* myeloperoxidase, *PR3* proteinase 3, *RBCs* red blood cells, *WBCs* white blood cells

Since an association of leukocyturia and glomerular damage in terms of crescent formation was detected, we next considered the fraction of glomeruli affected by BCR (classified into “focal” and “extensive”, with the latter meaning an increased glomerular affection with BCR by more than 50%, while both categories were indicated as fraction of total glomeruli per specimen). Extensive BCR correlated significantly with hematuria in ANCA GN (*p* = 0.005, *r* = 0.475, Fig. 3A, B), particularly attributed to PR3-ANCA GN (*p* = 0.015, *r* = 0.565, Fig. 3C, D). Moreover, leukocyturia positively correlated with focal BCR in ANCA GN (*p* = 0.025, *r* = 0.384, Fig. 3A, B) and PR3-ANCA GN (*p* = 0.003, *r* = 0.664, Fig. 3C, D), as confirmed by linear regression analysis. In contrast to these findings, no associations between leukocyturia and focal BCR (*p* = 0.944) or hematuria and extensive BCR (*p* = 0.271) were detected in MPO-ANCA GN (Fig. 3E, F).

### Leukocyturia is associated with intrarenal plasma cell infiltrates in PR3-ANCA GN

**Fig. 4:**
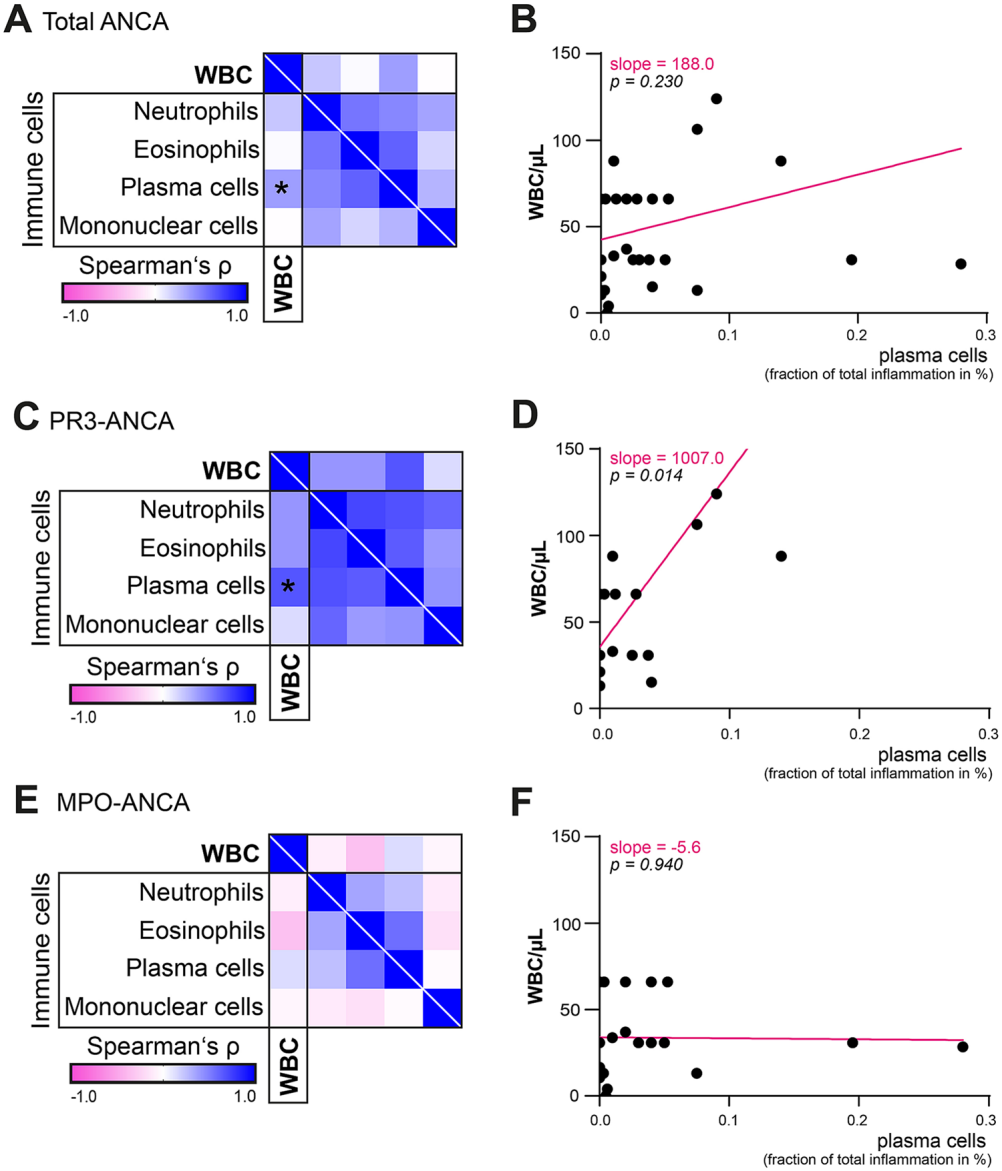
Association of leukocyturia with tubulointerstitial immune cell infiltration. **A**–**F** Fraction of total tubulointerstitial inflammation containing neutrophils, eosinophils, plasma cells and mononuclear cells was quantified. Heatmap visualizes mean values of Spearman’s ρ, asterisks represent *p* < 0.05, regression lines are shown in magenta. *ANCA* anti-neutrophil cytoplasmic antibody, *GN* glomerulonephritis, *MPO* myeloperoxidase, *PR3* proteinase 3, *WBCs* white blood cells

Based on our previously reported findings of the dependence of tubulointerstitial immune cell infiltration and the extent of BCR, we next analyzed the association of leukocyturia and tubulointerstitial immune cell infiltrations quantified as a fraction of total inflammation containing neutrophils, eosinophils, plasma cells and mononuclear cells (macrophages and lymphocytes) [22]. In the overall cohort of ANCA GN, a significant correlation between leukocyturia and plasma cell infiltration was identified (*p* = 0.019, *r* = 0.380, Fig. 4A, B), which was also observable in PR3-ANCA GN (*p* = 0.002, *r* = 0.664, Fig. 4C, D). In contrast to this, no correlation of leukocyturia and plasma cell infiltration was observed in MPO-ANCA GN (*p* = 0.577, Fig. 4E, F).

### Exclusion of potential confounders for leukocyturia in ANCA GN

**Fig. 5:**
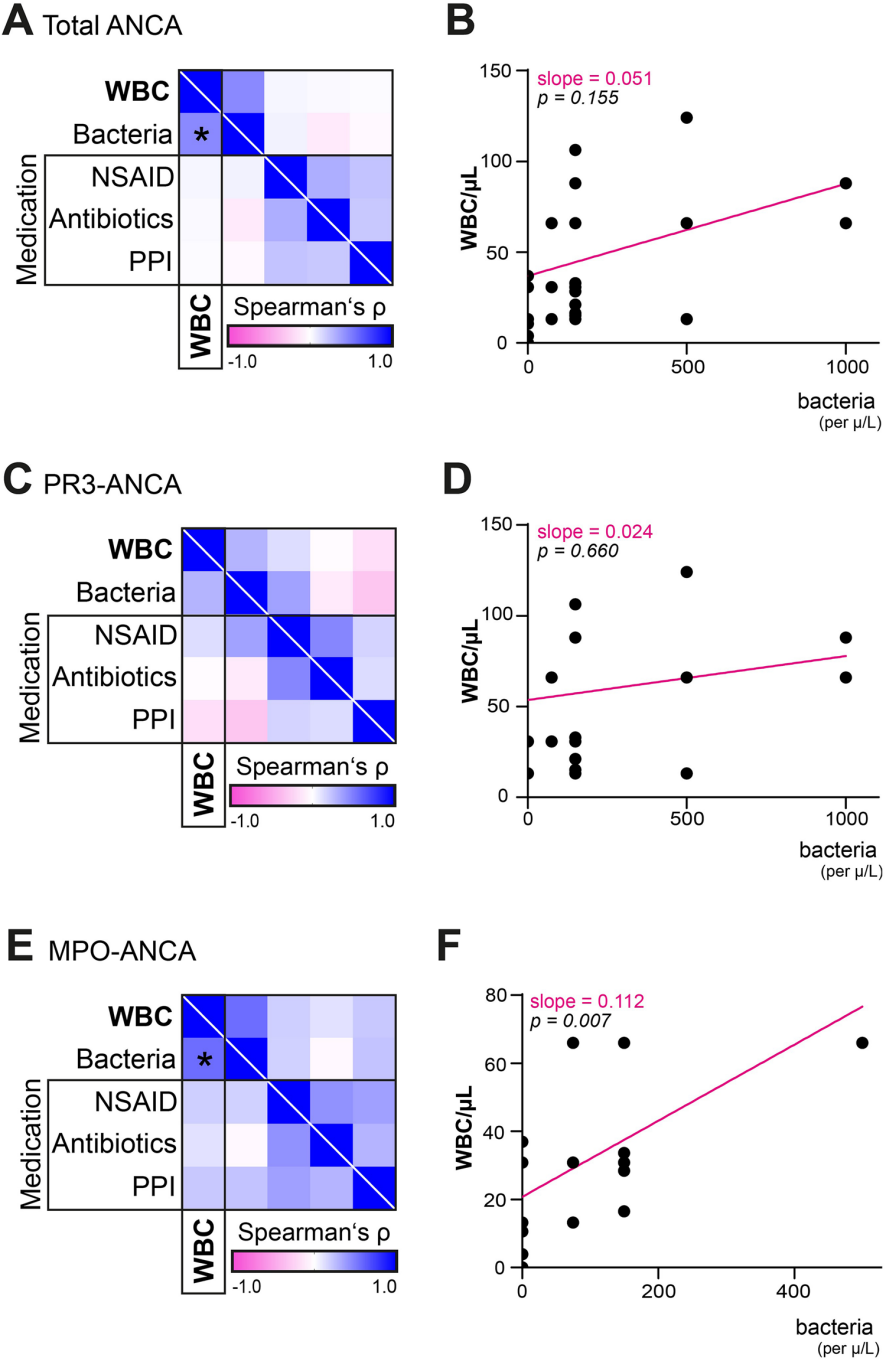
Correlation of leukocyturia with taken medication and bacteriuria. **A**–**F** WBCs indicate leukocyturia. Heatmap visualizes mean values of Spearman’s ρ, asterisks represent *p* < 0.05, linear regression of leukocyturia and bacteria, regression lines are shown in magenta. *ANCA* anti-neutrophil cytoplasmic antibody, *MPO* myeloperoxidase, *NSAID* non-steroidal anti-inflammatory drug, *PPI* proton-pump inhibitor, *PR3* proteinase 3, *WBCs* white blood cells

As a potential confounder due to acute interstitial nephritis manifesting as sterile leukocyturia, we finally analyzed the association of the intake of the most common acute interstitial nephritis-triggering medications, including antibiotics, non-steroidal anti-inflammatory drugs (NSAIDs), and proton-pump inhibitors (PPIs) with the presence of bacteriuria. We found no significant correlation between leukocyturia, bacteriuria or medication in the overall cohort of ANCA GN (NSAIDs: *p* = 0.827, antibiotics: *p* = 0.886, PPIs: *p* = 0.929, Fig. 5A, B), the PR3-ANCA GN cohort (NSAIDs: *p* = 0.571, antibiotics: *p* = 0.922, PPIs: *p* = 0.413, Fig. 5C, D) and in the MPO-ANCA GN cohort (NSAIDs: *p* = 0.426, antibiotics: *p* = 0.626, PPIs: *p* = 0.364, Fig. 5E, F). Another possible biasing effect due to differing kidney biopsy timing with respect to disease stages was also considered. Therefore, we compared the time to kidney biopsy after admission between the PR3-ANCA and the MPO-ANCA subgroup and found no significant difference (*p* = 0.5104, Supplemental Table 2). Moreover, comparison of the onset of symptoms before admission in both subgroups showed no significant difference (*p* = 0.1872, Supplemental Table 2). Finally, we compared laboratory parameters indicative of kidney injury and urinary WBCs in both subgroups, wherein significant differences of serum creatinine values became apparent (*p* = 0.0167, Supplemental Table 2) implying more severe kidney injury in MPO-ANCA, which was supported by group comparisons concerning the ARRS featuring significant differences (*p* = 0.0247, Supplemental Table 2). Spearman’s correlation regarding WBCs and kidney injury parameters showed no further significant associations (Supplemental Fig. 2). In summary, our data suggest no confounding effect of medication, bacteriuria, onset of symptoms, date of admission, or kidney biopsy on the occurrence of leukocyturia in the PR3-ANCA GN subgroup. A more severe extent of kidney damage was implied in the MPO-ANCA subgroup.

## Discussion

Herein we show that non-invasively detected urinary leukocytes and erythrocytes reflect different histopathological damage patterns in PR3-ANCA GN. First, the correlation of leukocyturia and hematuria was identified, wherein both were associated with markers of non selective glomerular damage. Second, non-invasive measurement of leukocyturia indicated focal (but not extensive) BCR specifically in PR3-ANCA GN, whereas hematuria correlated with extensive (but not focal) BCR. Third, leukocyturia was associated with interstitial fibrosis and an increased fraction of plasma cell infiltration in PR3-ANCA, whereas no such association was detectable in the MPO-ANCA GN cohort, thus implying distinct modes of kidney damage.

The unique contribution of B cells and plasma cells in renal autoimmunity is increasingly being recognized. From the reverse translational perspective, the therapeutic efficacy of rituximab, an agent directed against CD20-positive B cells, and bortezomib, a proteasome inhibitor targeting plasma cells, has repeatedly emphasized the pathophysiological relevance of B-lineage cells in many autoimmune diseases [23]. Originating from both B1- and B2-cell lineages, plasma cells follow terminal differentiation upon specific stimulation resulting in different, e.g., short- and long-lived subsets. The latter are also called memory plasma cells that pursue the main function of high-affinity antibody production and mainly reside in the bone marrow in healthy individuals. In chronic inflammatory states, other tissues can function as survival niches for memory plasma cells, where other cell types, such as basophils and eosinophils, maintain plasma cell recruitment by producing survival factors [23]. Moreover, immune cell infiltration reveals organizational clusters in ANCA GN that are often referred to as tertiary lymphoid organs or tertiary lymphoid structures (TLSs) [24]. These lymphocytic organizations are known to impair renal outcome in ANCA GN [24]. Based on observations that lymphocytic organizational clusters lack CD138-positive plasma cells in ANCA GN, the pathophysiological role of plasma cells was long underscored, whereas contrasting recent investigations from the field of cancer research highlight another function of plasma cells in TLS formation. In the context of B-cell-dependent tumor microenvironment, TLSs have been shown to promote B cell maturation towards IgG-producing plasma cells with the consequence of elevated immunotherapy-response rates in IgG-rich tumor masses [25]. Also, in stromal tumors such as ovarian cancer, a high association of TLSs, plasma cells and tumor-infiltrating T cells was identified and seen as a marker of anti-tumor immunity [26]. Antibody production of plasma cells is implicated in neutralizing their cognate antigens, activating the complement system, and inducing antibody-dependent cell toxicity, wherein plasma cells migrate in TLSs along fibroblastic routes [26]. In chronic inflammatory states, fibroblasts contribute to TLS formation [26]. Moreover, direct interleukin-dependent crosstalk between fibroblasts and plasma cells has been shown in psoriasis patients [27]. Still, the relevance of plasma cells in the pathophysiology of renal autoimmunity and interstitial fibrosis requires further investigation.

Considering that the triangulated interconnection of B-lineage cells is contributory to renal interstitial fibrosis, and that autoreactive PR3-positive B cells are pathophysiologically relevant in PR3-AAV, our data reveal an explicit degree of coherence since leukocyturia independently correlated with plasma cell infiltration, focal BCR and interstitial fibrosis in PR3-positive ANCA GN. We previously reported on the mechanistic concept of BCR linking severe glomerular damage to tubulointerstitial inflammation in ANCA GN [28]. An increased fraction of BCR-affected glomeruli, namely extensive BCR, correlated with tubulitis and interstitial inflammation. Moreover, we recently showed that tubulointerstitial immune cell involvement features a distinct associative pattern depending on the extent of BCR [10]. While extensive BCR was mainly associated with intrarenal infiltration of neutrophils, focal BCR featured a more versatile picture of immune cell replenishment featured by plasma cell and neutrophilic infiltrates [10]. Furthermore, we previously showed that plasma cell and neutrophilic infiltrations are associated with interstitial fibrosis in PR3-ANCA GN [22].

The established mechanistic comprehension of AAV regards, in case of acute injury phase, neutrophils to predominately act as effector leukocytes that spearhead inflammation, while in the so-called “response-to-injury” phase mainly lymphocytes, monocytes and macrophages contribute to damage mediation [29, 30]. Asynchronicity of ongoing multiple acute inflammatory microlesions, each following a stereotypical process from acute to chronic, was pointed out [29, 30]. Against the background of these notions, the finding of leukocyturia coincidentally associating with focal BCR and plasma cell infiltration, while synchronically exposing no correlation with active tubulointerstitial inflammation, might reflect a smoldering state of intrarenal inflammation that partially predisposes transition into an aggravated form of kidney damage and partially represents ongoing damage mediation. Adding up to this notion, we found no correlation of leukocyturia and extensive BCR in the MPO-ANCA subgroup, wherein the extent of kidney damage appeared to be more severe in the MPO-ANCA subgroup as implied by comparisons of the ARRS groups. Therefore, we cannot exclude that leukocyturia might also occur in milder forms of kidney injury in MPO-ANCA GN. In this context, we addressed potential biasing effects occurring due to differing timing of renal biopsy during the ongoing inflammatory flare in renal AAV since clinical management is often labeled by affected patients frequently exposing an oligo-symptomatic stage that pioneers acute disease stages and thereby influencing mutable timings of kidney biopsy. Here, we found no significant differences between the PR3-ANCA and MPO-ANCA subgroups regarding onset of symptoms before admission and time to renal biopsy.

This study has several limitations. First, this is a retrospective study requiring independent validation. Second, the limited patient number may reduce generalizability of our findings. Third, identification of urinary leukocyte phenotype by flow cytometry would be of high interest. Fourth, also MPO-ANCA GN might feature leukocyturia during milder forms of kidney injury. Nevertheless, we here expand our current knowledge providing evidence that leukocyturia and hematuria enable non-invasive differentiation between BCR severity specifically in PR3-ANCA GN.

## Supplementary Information

Below is the link to the electronic supplementary material.

Supplementary file1 (PDF 790 kb)

## Data Availability

Deidentified data are available on reasonable request from the corresponding author.
