# Supplementary material for: Leukocyturia and hematuria enable non-invasive differentiation of Bowman’s capsule rupture severity in PR3-ANCA glomerulonephritis
Source: J Nephrol. 2022 Dec 21;36(3):799–808. doi: 10.1007/s40620-022-01486-8 (PMC10090024; doi:10.1007/s40620-022-01486-8)
Supplement: Supplementary file 1 — Supplementary file1 (PDF 790 kb) [file 40620_2022_1486_MOESM1_ESM.pdf]

Leukocyturia and hematuria enable non-invasive differentiation between extent of Bowman's capsule rupture in PR3-ANCA glomerulonephritis

Supplemental Material

Eva Baier[1], Ingmar Alexander Kluge[2], Samy Hakroush[2,3], Désirée Tampe[1], Björn Tampe[1*]

[1]*Department of Nephrology and Rheumatology, University Medical Center Göttingen, Germany*

[2]*Institute of Pathology, University Medical Center Göttingen, Germany*

[3]*SYNLAB Pathology Hannover, SYNLAB Holding Germany, Augsburg, Germany*

[*]*Corresponding author*

Running title: Leukocyturia and hematuria in ANCA GN

Corresponding author

Björn Tampe, MD

Department of Nephrology and Rheumatology

University Medical Center Göttingen

Robert Koch Street 40

37075 Göttingen, Germany

Email: bjoern.tampe@med.uni-goettingen.de

Supplemental Table 1. Clinical and histopathological parameters of the total ANCA GN, MPO-ANCA GN and PR3-ANCA GN cohorts.

	Total cohort (n=42)	MPO-ANCA (n=21)	PR3-ANCA (n=21)
Clinical data			
Female sex – no. (%)	19 (45.2)	6 (28.6)	13 (61.9)
Age – years	61.7 ± 14.9	60.4 ± 13.5	62.8 ± 16.4
BP at biopsy (systolic/diastolic) - mmHg	129 ± 13/74 ± 11	128 ± 14/72 ± 13	130 ± 11/ 7 ± 7
BMI – kg/m^2	26 ± 4	27 ± 4	24 ± 3
ANCA subtype			
MPO – no (%)	21 (50)	21 (50)	0 (0)
PR3 – no (%)	21 (50)	0 (0)	21 (50)
Double-positive – no (%)	0 (0)	0 (0)	0 (0)
Treatment			
PEX – no (%)	17 (40.5)	9 (42.9)	8 (38.1)
RTX – no (%)	21 (50)	11 (52.4)	10 (47.6)
CYC – no (%)	24 (57.1)	10 (47.6)	14 (66.7)
steroid pulse – no (%)	27 (64.3)	12 (57.1)	15 (71.4)
AAV relapse – no. (%)	8 (19)	5 (23.8)	3 (14.3)
BVAS – points	18 ± 4.4	17.3 ± 4.4	18.6 ± 4.3
Death – no (%)	3 (7.1)	1 (4.8)	2 (9.5)
Onset of symptoms before admission – days	45.9 ± 82.5	53.0 ± 113.6	39.1 ± 35.4
Extrarenal manifestations			
Hemoptysis – no. (%)	6 (14.3)	3 (14.3)	3 (14.3)
Lung – no. (%)	23 (54.8)	11 (52.4)	12 (57.1)
Sinus – no. (%)	9 (21.4)	1 (4.8)	8 (38.1)
Skin – no. (%)	7 (16.7)	3 (14.3)	4 (19.0)
Kidney function			
Creatinine at admission	2.9 ± 2.1	3.7 ± 2.3	2.1 ± 1.6
eGFR at admission	40.1 ± 36.1	29.9 ± 30.8	50.3 ± 38.7
BUN at admission	45.5 ± 25.9	49.0 ± 24.6	41.4 ± 27.4
CKD stage before admission			
G1 – no (%)	11 (26.2)	5 (23.8)	6 (28.6)
G2 – no (%)	16 (38.1)	7 (33.3)	9 (42.9)
G3 a/b – no (%)	2 (4.8)/ 3 (7.1)	0 (0)/ 2 (9.5)	2 (9.5)/ 1 (4.8)
G4 – no (%)	3 (7.1)	3 (14.3)	0 (0)
G5 – no (%)	2 (4.8)	1 (4.8)	1 (4.8)
KRT during hospital stay – no (%)	13 (31.0)	7 (33.3)	6 (28.6)
Kidney biopsy after admission – days	7.0 ± 4.5	7.1 ± 4.0	6.8 ± 5.1
Indication for kidney biopsy: a/b/c/d – no (%)	25/21/18/3	10/10/8/1	15/11/10/2
Indication for kidney biopsy (AAV relapse): a/b/c/d	3/2/0/3	2/0/0/1	1/2/0/2
Medication at time of biopsy			
NSAID – no. (%)	3 (7.1)	3 (14.3)	1 (4.8)
Antibiotics – no. (%)	9 (21.4)	5 (23.8)	4 (19)
PPI – no. (%)	27 (64.3)	12 (57.1)	15 (71.4)
Urinary sediment			
White blood cells – particles/µL	46.3 ± 53.4	32.6 ± 21.7	59.9 ± 70.5
Red blood cells – particles/µL	151.9 ± 255.8	186 ± 328	117.8 ± 155.2
Squamous epithelial cells – particles/µL	1.4 ± 4.1	0.9 ± 3.4	1.9 ± 4.8
Hyaline casts – particles/µL	1.5 ± 4.2	0.9 ± 3.4	2.2 ± 5.1
Granular casts – particles/µL	1.5 ± 4.2	1.8 ± 4.6	1.1 ± 3.8
Bacteria – particles/µL	183.9 ± 234.9	106 ± 110.7	261.9 ± 296.8
Urinary dipstick			
pH – dimensionless value	5.6 ± 0.8	5.6 ± 0.7	5.6 ± 1
White blood cells – particles/µL	51.8 ± 130.1	31.5 ± 107.9	73.1 ± 150
Red blood cells – particles/µL	89.3 ± 101.3	87.9 ± 97.1	90.8 ± 108
Hyaline casts – particles/µL	1.5 ± 2.4	1 ± 2.2	2 ± 2.7
Spec. weight – dimensionless value	1 ± 0.006	1 ± 0.003	1 ± 0.007
Proteinuric findings			
uPCR – mg/g creatinine	1749 ± 2082	2525.5 ± 2728.6	1035 ± 748
uACR – mg/g creatinine	940 ± 1393	1468.1 ± 1842.3	454.2 ± 417
α_1-microglobulin – mg/g creatinine	123.3 ± 162.5	116.3 ± 109.4	129.7 ± 201.7
IgG – mg/g creatinine	127.2 ± 172.1	186 ± 220.9	70.8 ± 76.2
α_2-macroglobulin – mg/g creatinine	9 ± 10.4	12.5 ± 13.7	5.8 ± 4.4
ANCA classification by Berden et al.			
Crescentic class – no. (%)	14 (33.3)	8 (38.1)	6 (28.6)
Focal class – no. (%)	20 (47.6)	7 (33.3)	13 (61.9)
Mixed class – no. (%)	5 (11.9)	3 (4.8)	2 (9.5)
Sclerotic class – no. (%)	3 (7.1)	3 (4.8)	0 (0)
ARRS by Brix et al.			
High risk – no. (%)	8 (19.0)	7 (33.3)	1 (4.8)
Medium risk – no. (%)	17 (40.5)	9 (42.9)	8 (38.1)
Low risk – no. (%)	17 (40.5)	5 (23.8)	12 (57.1)
Glomerular lesions			
Normal glomeruli – % of total	48.9 ± 29.2	39.4 ± 30.8	57.6 ± 25.2
Necrotic glomeruli – % of total	25 ± 28.6	25.7 ± 31.1	24.3 ± 26.9
Crescentic glomeruli – % of total	36.3 ± 28.7	41.2 ± 31	31.7 ± 26.1
Sclerotic glomeruli – % of total	16.9 ± 25.4	24.2 ± 33.3	10.2 ± 12.3

Bowman's capsule rupture			
Focal BCR – % of total	28.4 ± 33.5	30.8 ± 33.4	26.3 ± 34.2
Extensive BCR – % of total	5.1 ± 13.9	2 ± 4.8	7.8 ± 18.2
Banff scoring			
i – Banff lesion score	0.2 ± 0.4	0.2 ± 0.4	0.1 ± 0.4
t – Banff lesion score	0.7 ± 0.9	0.8 ± 0.8	0.6 ± 0.9
v – Banff lesion score	0.5 ± 1	0.7 ± 1.2	0.4 ± 0.9
g – Banff lesion score	1.7 ± 0.9	1.6 ± 1.1	1.8 ± 0.8
ci – Banff lesion score	1.3 ± 0.9	1.6 ± 0.8	1 ± 0.8
ct – Banff lesion score	1.3 ± 0.7	1.6 ± 0.7	1 ± 0.6
ptc – Banff lesion score	0.1 ± 0.3	0.7 ± 1	0.1 ± 0.3
ti – Banff lesion score	0.8 ± 0.8	1.1 ± 0.7	0.6 ± 0.9
i-IFTA – Banff lesion score	1.8 ± 1.4	1.9 ± 1	1.6 ± 1.3
t-IFTA – Banff lesion score	0.7 ± 0.1	0.8 ± 0.5	0.6 ± 0.5
Tubular injury lesions			
Tubular dilatation – score	2.5 ± 1.3	2.9 ± 1.2	2.2 ± 1.3
Tubular vacuolization – score	0.2 ± 0.4	0.2 ± 0.4	0.2 ± 0.4
Tubular cellular casts – score	0.8 ± 0.8	0.8 ± 0.6	0.7 ± 1
Tubular RBC casts – score	0.5 ± 0.6	0.5 ± 0.7	0.5 ± 0.6
Tamm Horsfall protein – score	0.8 ± 0.8	0.9 ± 0.9	0.6 ± 0.6
Immune cell infiltration			
Neutrophils – % of total inflammation	0.9 ± 1.6	0.5 ± 1	1.3 ± 2
Eosinophils – % of total inflammation	0.6 ± 1.4	0.6 ± 1.7	0.5 ± 1.1
Plasma cells – % of total inflammation	3.2 ± 5.6	4.1 ± 7	2.4 ± 3.8
Mononuclear cells – % of total inflammation	9.8 ± 9.3	10.5 ± 7.8	9 ± 10.6

Continuous variables are expressed as mean ± standard deviation, categorical variables are presented as frequency and percentage of total. CKD stages based on KDIGO guidelines. Abbreviations: a, acute kidney injury; AAV, ANCA-associated vasculitis; ANCA, anti-neutrophil cytoplasmic antibody; ARRS, ANCA renal risk score; b, progressive proteinuria; BP, blood pressure; BMI, body mass index; BUN, blood urea nitrogen; BVAS, Birmingham Vasculitis Activity Score; c, hematuria; CKD, chronic kidney disease; CKD-EPI, chronic kidney disease epidemiology collaboration; *ct*, tubular atrophy; CYC, Cyclophosphamide; d, follow-up biopsy; eGFR, estimated glomerular filtration rate (CKD-EPI); *g*, glomerulitis;*i*, interstitial inflammation; *i-IFTA*, inflammation in IFTA;KRT, kidney replacement therapy; MPO, myeloperoxidase; no., number; NSAID, nonsteroidal anti-inflammatory drug; PPI, proton pump inhibitor; PR3, proteinase 3; *ptc*, peritubular capillaritis; RBC, red blood cell; RTX, Rituximab;*t*, tubulitis; *ti*, total inflammation; *t-IFTA*, tubulitis in IFTA; *v*, intimal arteritis..

Supplemental Table 2. Group comparisons of clinical and histopathological parameters of the MPO-ANCA and PR3-ANCA subgroups.

	MPO-ANCA (n=21)	PR3-ANCA (n=21)	*p* value
Clinical data			
Age – years	60.4 ± 13.5	62.8 ± 16.4	*0.1617*
BP at biopsy (systolic/diastolic) - mmHg	128 ± 14/ 72 ± 13	130 ± 11/ 77 ± 7	*0.69/ 0.15*
BMI – kg/m^2	27 ± 4	24 ± 3	*0.0736*
BVAS – points	17.3 ± 4.4	18.6 ± 4.3	*0.1272*
Onset of symptoms before admission – days	53.0 ± 113.6	39.1 ± 35.4	*0.1872*
Kidney function			
Creatinine at admission – mg/dL	3.7 ± 2.3	2.1 ± 1.6	***0.0167***
eGFR at admission – mL/min.	29.9 ± 30.8	50.3 ± 38.7	*0.0528*
BUN at admission -	49.0 ± 24.6	41.4 ± 27.4	*0.2468*
Kidney biopsy after admission – days	7.1 ± 4.0	6.8 ± 5.1	*0.5104*
Urinary sediment			
White blood cells – particles/µL	32.6 ± 21.7	59.9 ± 70.5	*0.1883*
Red blood cells – particles/µL	186 ± 328	117.8 ± 155.2	*0.3675*
Squamous epithelial cells – particles/µL	0.9 ± 3.4	1.9 ± 4.8	*0.5977*
Hyaline casts – particles/µL	0.9 ± 3.4	2.2 ± 5.1	*0.5692*
Granular casts – particles/µL	1.8 ± 4.6	1.1 ± 3.8	*0.9999*
Bacteria – particles/µL	106 ± 110.7	261.9 ± 296.8	*0.0662*
Urinary dipstick			
pH – dimensionless value	5.6 ± 0.7	5.6 ± 1	*0.6899*
White blood cells – particles/µL	31.5 ± 107.9	73.1 ± 150	*0.0941*
Red blood cells – particles/µL	87.9 ± 97.1	90.8 ± 108	*0.5834*
Hyaline casts – particles/µL	1 ± 2.2	2 ± 2.7	*0.9999*
Spec. weight – dimensionless value	1 ± 0.003	1 ± 0.007	***0.0073***
Proteinuric findings			
uPCR – mg/g creatinine	2525.5 ± 2728.6	1035 ± 748	***0.0294***
uACR – mg/g creatinine	1468.1 ± 1842.3	454.2 ± 417	***0.0117***
α_1-microglobulin – mg/g creatinine	116.3 ± 109.4	129.7 ± 201.7	*0.5837*
IgG – mg/g creatinine	186 ± 220.9	70.8 ± 76.2	***0.0336***
α_2-macroglobulin – mg/g creatinine	12.5 ± 13.7	5.8 ± 4.4	***0.0293***
ANCA classification by Berden et al.			
Crescentic class – no. (%)	8 (38.1)	6 (28.6)	
Focal class – no. (%)	7 (33.3)	13 (61.9)	
Mixed class – no. (%)	3 (4.8)	2 (9.5)	*0.1520*
Sclerotic class – no. (%)	3 (4.8)	0 (0)	
ARRS by Brix et al.			
High risk – no. (%)	7 (33.3)	1 (4.8)	
Medium risk – no. (%)	9 (42.9)	8 (38.1)	
Low risk – no. (%)	5 (23.8)	12 (57.1)	***0.0247***
Glomerular lesions			
Normal glomeruli – % of total	39.4 ± 30.8	57.6 ± 25.2	***0.0125***
Necrotic glomeruli – % of total	25.7 ± 31.1	24.3 ± 26.9	*0.9343*
Crescentic glomeruli – % of total	41.2 ± 31	31.7 ± 26.1	*0.2192*
Sclerotic glomeruli – % of total	24.2 ± 33.3	10.2 ± 12.3	*0.4946*

Continuous variables are expressed as mean ± standard deviation, categorical variables are presented as frequency and percentage of total. Group comparisons between the PR3- and MPO-ANCA group were conducted. Unpaired student's t-test was performed for comparison of two normally distributed groups, Mann-Whitney test for comparisons of two non-normally distributed groups. Nonparametric between-group-comparisons were performed with Pearson's Chi-square test.
Abbreviations: ANCA, anti-neutrophil cytoplasmic antibody; ARRS, ANCA renal risk score; BP, blood pressure; BMI, body mass index; BUN, blood urea nitrogen; BVAS, Birmingham Vasculitis Activity Score; CKD-EPI, chronic kidney disease epidemiology collaboration; eGFR, estimated glomerular filtration rate (CKD-EPI); MPO, myeloperoxidase; no., number; PR3, proteinase 3.

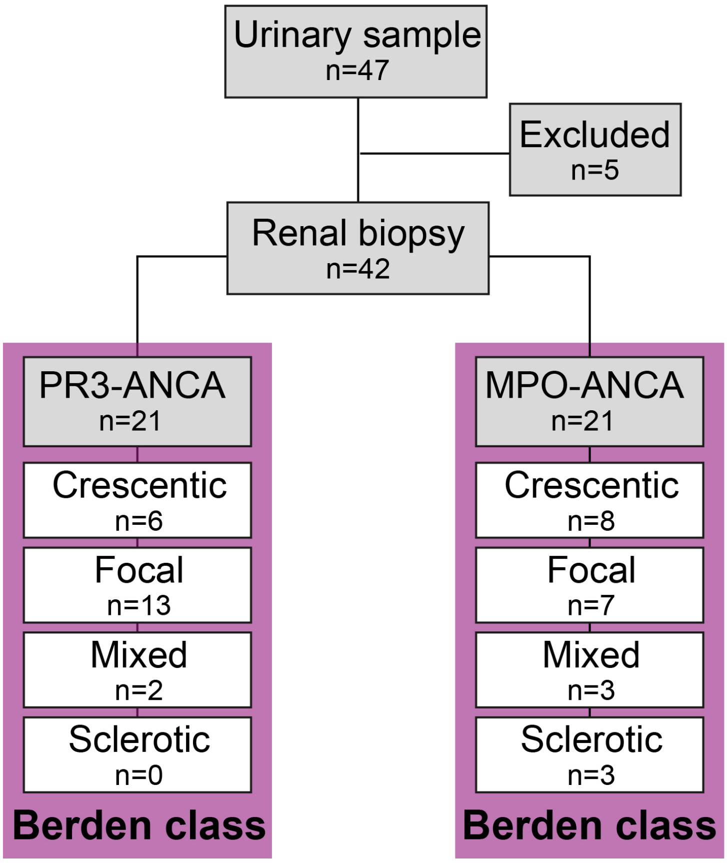

Supplemental Figure 1. STROBE flow chart of patient disposition included in this study. Histopathological classification according to Berden, "Excluded" due to coincident bacteriuria and leukocyturia in the respective urine culture.

Abbreviations: ANCA, anti-neutrophil cytoplasmic antibody; MPO, myeloperoxidase; PR3, proteinase 3; STROBE, Strengthening the Reporting of Observational Studies in Epidemiology.

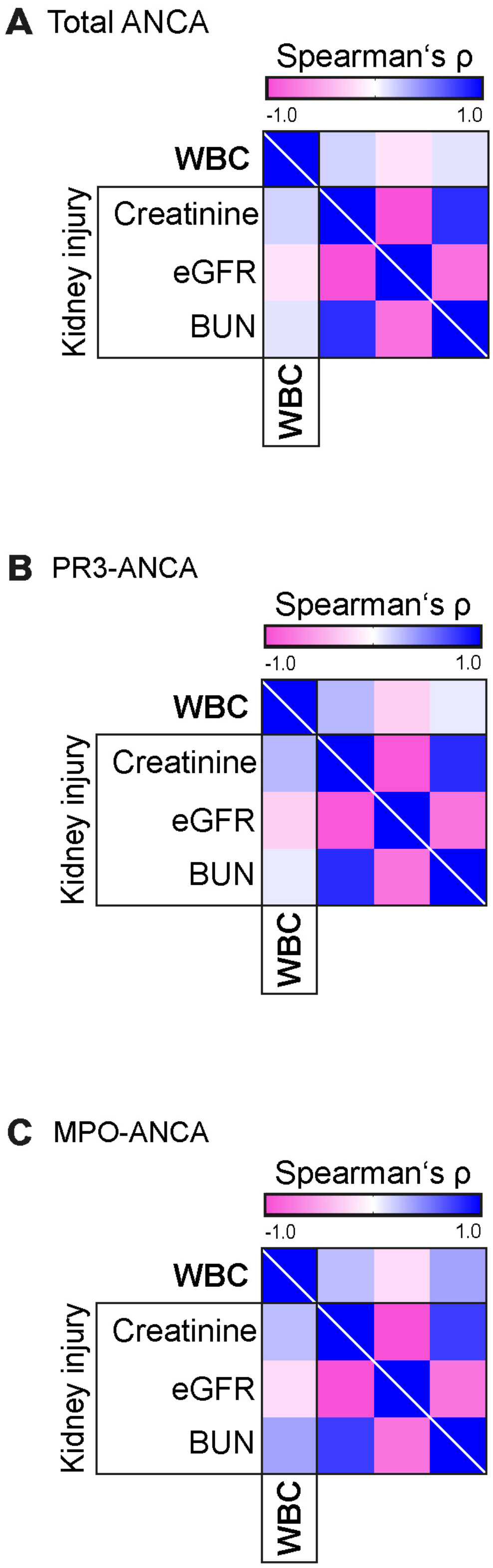

Supplemental Figure 2. Correlation of leukocyturia with laboratory parameters indicative of kidney injury. (**A-C**) WBC indicate leukocyturia. Heatmap visualizes mean values of Spearman’s ρ, asterisks represent $p<0.05$.

Abbreviations: ANCA, anti-neutrophil cytoplasmic antibody; BUN, blood urea nitrogen; CKD-EPI, Chronic Kidney Disease Epidemiology Collaboration; eGFR, estimated glomerular filtration rate (CKD-EPI); MPO, myeloperoxidase; PR3, proteinase 3; WBC, white blood cells.
